# Case Report: Cytoreductive Surgery and Hyperthermic Intraperitoneal Chemotherapy Application in Intraperitoneally Disseminated Inflammatory Myofibroblastic Tumor and in the Youngest Patient in the World: New Indication and Modification of Technique

**DOI:** 10.3389/fsurg.2021.746700

**Published:** 2021-10-12

**Authors:** Hanna Garnier, Maciej Murawski, Tomasz Jastrzebski, Katarzyna Pawinska-Wasikowska, Walentyna Balwierz, Katarzyna Sinacka, Wojciech Gorecki, Ewa Izycka-Swieszewska, Piotr Czauderna

**Affiliations:** ^1^Department of Surgery and Urology for Children and Adolescents, Medical University of Gdansk, Gdańsk, Poland; ^2^Department of Surgical Oncology, Medical University of Gdansk, Gdańsk, Poland; ^3^Department of Pediatric Oncology and Hematology, Jagiellonian University Medical College, Krakow, Poland; ^4^2nd Radiology Department, Medical University of Gdansk, Gdańsk, Poland; ^5^Department of Pediatric Surgery, University Children's Hospital, Kraków, Poland; ^6^Department of Pathology and Neuropathology, Medical University of Gdansk, Gdańsk, Poland

**Keywords:** HIPEC (heated intraperitoneal chemotherapy), cytoreductive surgery, inflammatory myofibroblastic tumor, pediatric oncology, oncological surgical treatment

## Abstract

**Introduction:** Peritoneal metastases occur in cancers that spread to the peritoneal cavity and indicate the advanced stage of the disease. In children they are mainly seen in sarcomas, Gastrointestinal Stromal Tumors and primary disseminated ovarian tumors. Inflammatory Myofibroblastic Tumor (IMT) is a very rare lesion, characterized by an unpredictable clinical course. The absorption of chemotherapeutic agents through the peritoneal-plasma barrier (PPB) is minimized, thus HIPEC procedure limits the systemic exposure to chemotherapy and permits the administration of its higher doses. The main purpose of HIPEC is to remove the visible macroscopic disease in order to achieve complete cytoreduction (CRS).

**HIPEC Procedure in Children:** Several papers deal with the CRS and HIPEC in children and adolescents, however pediatric experience is still limited. Thus far, the HIPEC procedure has been carried out on patients over 2 years old. The most common indication for the surgery and the best outcome was experienced by patients with desmoplastic small round cell tumor (DSRCT). Most patients received intraperitoneal cisplatin.

**HIPEC Modification:** A 5-month-old infant was admitted to the Department of Pediatric Oncology due to the abdominal distention and blood in the stool. The Computed Tomography (CT) revealed a solid-cystic mass in the right abdominal area. The primary tumor and numerous peritoneal metastasis were removed and the Inflammatory Myofibroblastic Tumor (IMT) was diagnosed. The patient underwent subsequently CRS and modified HIPEC procedure. To avoid overheating of the infant, the intraperitoneal normothermic chemoperfusion was performed. Due to the low body weight a modified dosage of intraperitoneal doxorubicin was used. The child underwent standard postoperative chemotherapy and received crizotinib therapy. At 12 months follow-up since treatment completion the patient remains in complete remission. To our knowledge this is the youngest patient, the only infant and the first pediatric patient with IMT who underwent the modified HIPEC procedure in the world.

**Conclusions:** CRS and HIPEC is technically possible also in infants. For its safe course patients selection and technique modification are necessary. Use of HIPEC should be also considered in intraperitoneally disseminated IMT. A complete cytoreductive surgery as the first HIPEC step seems to be the key factor in survival.

## Introduction

Frequency of peritoneal metastasis of cancer or sarcoma in children still remains unknown. Peritoneal involvement is mainly seen in sarcomas (i.e., Desmoplastic Small Round Cell Tumor DSRCT, rhabdomyosarcoma, leiomyosarcoma, liposarcoma, etc.), GastroIntestinal Stromal Tumors (GIST), primary disseminated ovarian tumors (i.e., yolk sac tumor, Sertoli and Leydig cell tumors, ovarian carcinomas, etc.) (1–3). Peritoneal metastases involve cancers that spread to the peritoneal cavity and usually indicate an advanced stage of the disease.

Inflammatory Myofibroblastic Tumor (IMT), also called inflammatory pseudotumor, is a very rare pulmonary or extra-pulmonary lesion, characterized by an unpredictable clinical course. It can be benign, malignantly transformed, recurrent, or even metastasize. These tumors are very difficult to distinguish from other neoplasms and a detailed histologic analysis is required to establish the diagnosis. The pathogenesis of this disease remains unknown, but some IMTs have altered anaplastic lymphoma kinase (ALK) expression mostly resulting from rearrangement of the ALK gene and its fusion with other genes such as: TPM3-ALK, TPM4-ALK, and CLTC-ALK (4). These tumors consist of spindle-shaped myofibroblastic cells accompanied by inflammatory infiltration of plasma cells, lymphocytes, and eosinophils. IMT is characterized by a low mitotic index, no atypical division figures, necrosis, nuclear atypia, and above all, it seems not to spread through blood vessels (5). IMT can be a result of genetic mutation, or secondary to infectious or autoimmune disease. The treatment of choice is surgical resection of the lesion and subsequent chemo or radiotherapy, however due to the rare nature of IMT, proper guidelines have yet to be established. An aggressive surgical management is usually necessary due to the lack of other effective treatment. Due to the fact that the biology of myofibroblastic hyperplasia remains unpredictable, further observation of patients after surgery is necessary (6, 7).

The first detailed description of cytoreductive procedures within the peritoneum are found in Sugarbaker's work based on adults (8). With this early promising data, the interest promptly spread throughout the medical world in hope of finding better outcomes for oncological patients. Within the years several studies in animals demonstrated prolonged survival in groups receiving hyperthermic intraperitoneal chemotherapy (HIPEC) (9, 10). The absorption of chemotherapeutic agents through the peritoneal-plasma barrier (PPB) is minimized, thus HIPEC procedure limits the systemic exposure to chemotherapy and permits the administration of its higher doses. The main purpose of HIPEC is to remove the visible macroscopic disease with complete cytoreduction (CRS) and exposed the remnant lesions to intraperitoneal chemotherapy.

## HIPEC and Cytoreductive Surgery in Children

**Figure 1:**
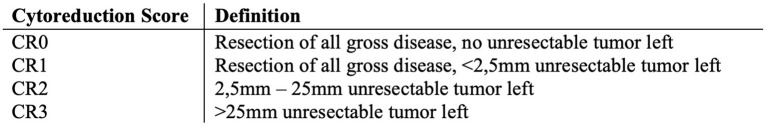
CR Scale. Completeness of CRS (11).

The possibility of complete (CR0) or near complete (CR1) cytoreduction is of key importance in selecting a patient for the CRS and HIPEC procedure. Patient's survival depends primarily on the completeness of cytoreduction measured by the CR Scale (Figure 1). The inability to obtain macroscopic clearance at resection (CR0 or CR1) may result in a decision to withdraw from the HIPEC procedure and initiate palliative treatment (12). Other important eligibility criterion is lack of distant metastases. Hayes-Jordan et al. (13) proved that no disease outside the abdomen at the time of surgery ensures the best outcome (disease-free interval 37.9 vs. 14.3 months). Otherwise, liver metastases do not exclude patients from the CRS-HIPEC procedure providing it is possible to either resect them at the time of surgery or treat them with radiation, or radiofrequency ablation (14, 15). Normal kidney function also seems to play a crucial role in qualifying the child for the procedure. According to the Owusu-Agyemang et al. study (16), to avoid renal toxicity during the CRS-HIPEC procedure, especially with cisplatin, it is important to maintain the urine output at an average of 3 ml/kg/h and the fluid administration must oscillate at an average rate of 9 ml/kg/h. The relative selecting criteria for CRS+HIPEC are as follows: a minimum interval of 4 weeks from the last radiotherapy or chemotherapy and an interval of more than 4 months from the last HIPEC procedure, life expectancy of more than 6 weeks, and normal liver function (17). It also seems that CRS+HIPEC is more effective and increases the survival rate of children with stable disease or partial remission after prior chemical treatment (12).

The HIPEC and CRS procedure can be performed in two ways: opened (Coliseum) and closed technique (18). A significant advantage of the open technique is that it allows a uniform drug distribution within the peritoneal cavity. The disadvantage of this technique, however, is the heat loss of the perfusion fluid and the potential risk of contamination of the operating field. The closed technique, contrarily, is associated with uneven distribution of the chemotherapeutics but eliminates exposure of the operating team to the cytotoxic drugs. Furthermore, many authors have observed that it provides more stable intraoperative conditions, making it the most relevant choice for pediatric patients (19, 20). Lotti et al. (21) in their study drew attention to the usage of laparoscopy during the HIPEC procedure. It combines the advantages of both, open and closed techniques and could be an interesting alternative for children.

**Figure 2:**
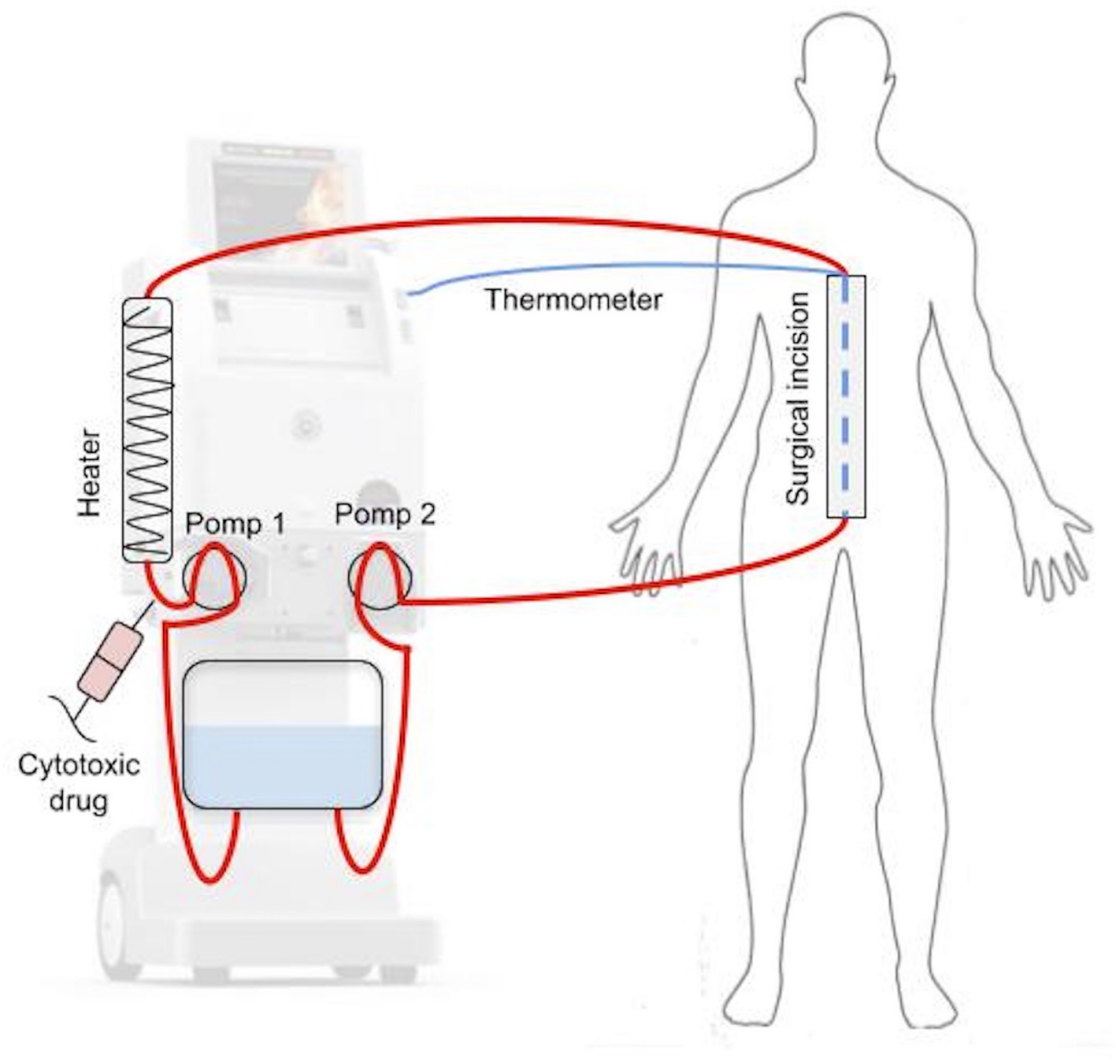
Schematic overview of HIPEC.

During HIPEC (Figure 2), after CRS phase, four drains are inserted into the peritoneal cavity: two delivering and two receiving the cytotoxic drug. Each of them is equipped with a thermometer to measure the temperature of the fluid entering and exiting the peritoneal cavity. Additionally, the temperature is usually measured in the sub-diaphragmic area and in the pelvis. Central temperature is measured with a temperature sensor located either in the esophagus or in the pulmonary artery. After insertion of the drains, perfusion fluid is administrated, usually Ringer's lactate, sodium chloride 0.9 or 5% glucose solution, depending on the anticancer drug used. The volume of fluid administrated ranges from 0.5 to 4 l and it is heated to 41–45°C. When the target temperature is reached, cytotoxic drugs are administrated. The perfusion time ranges between 30 and 90 min. After this period, the cytotoxic drugs are removed, and ~3 l of clean perfusion fluid is administrated to rinse the peritoneal cavity. The duration of CRS + HIPEC ranges usually from 4 to 10 h (22, 23).

Intraperitoneal administration of chemotherapy maximizes the chemotherapeutic dose delivered to peritoneal lesions while minimizing systemic toxicity. The most commonly used intraperitoneal agents are cisplatin (100 mg/m^2^) and doxorubicin (30 mg/m^2^). However, use of other cytostatics, such as mitomycin C, oxaliplatin, carboplatin, 5-FU, or taxanes has also been described (24).

## A Case Report and HIPEC Procedure Modification

**Figure 3:**
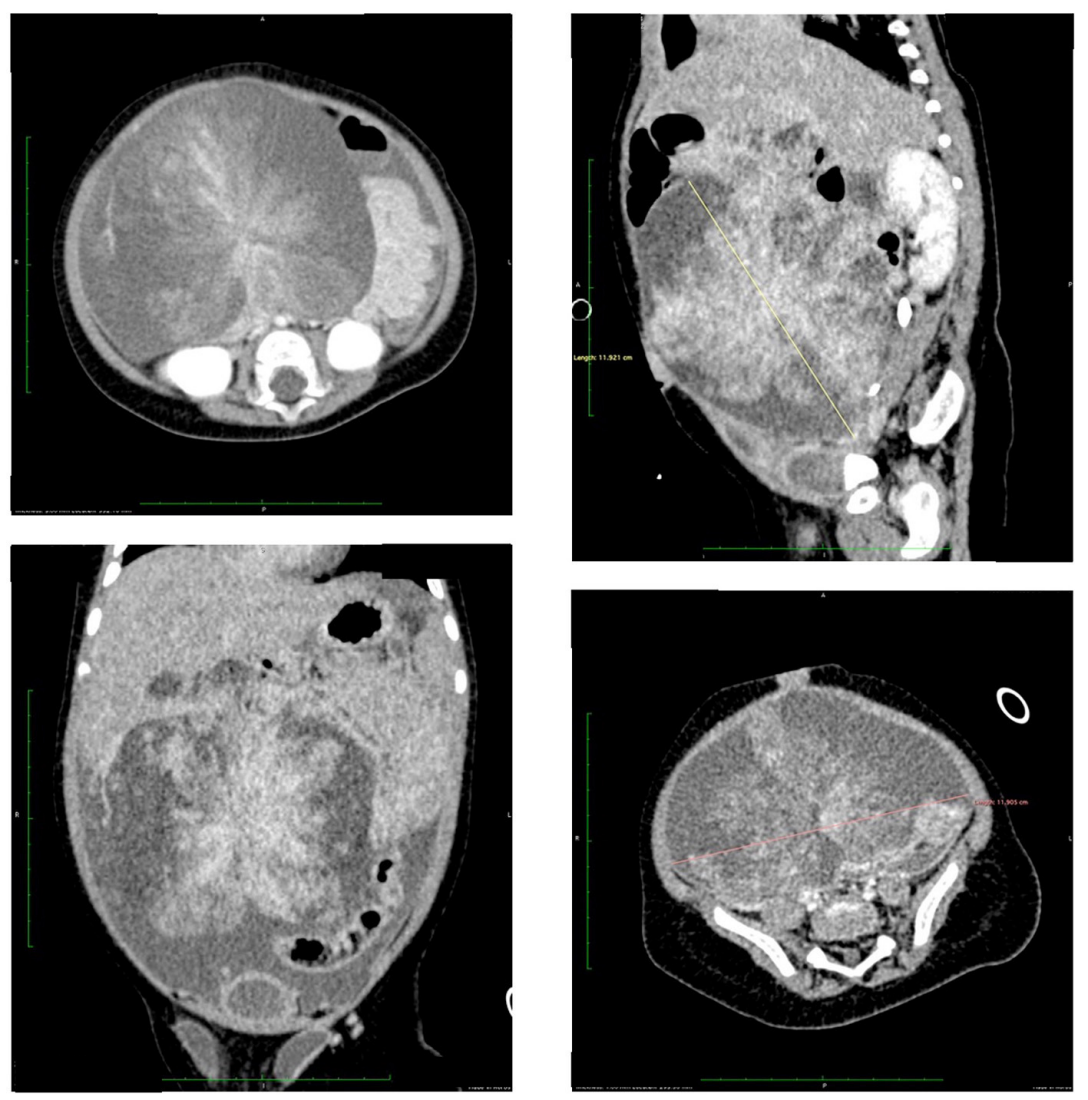
Pre-operative CT scan: a solid-cystic mass (113 × 98 × 103 mm) in the right abdominal area.

**Figure 4:**
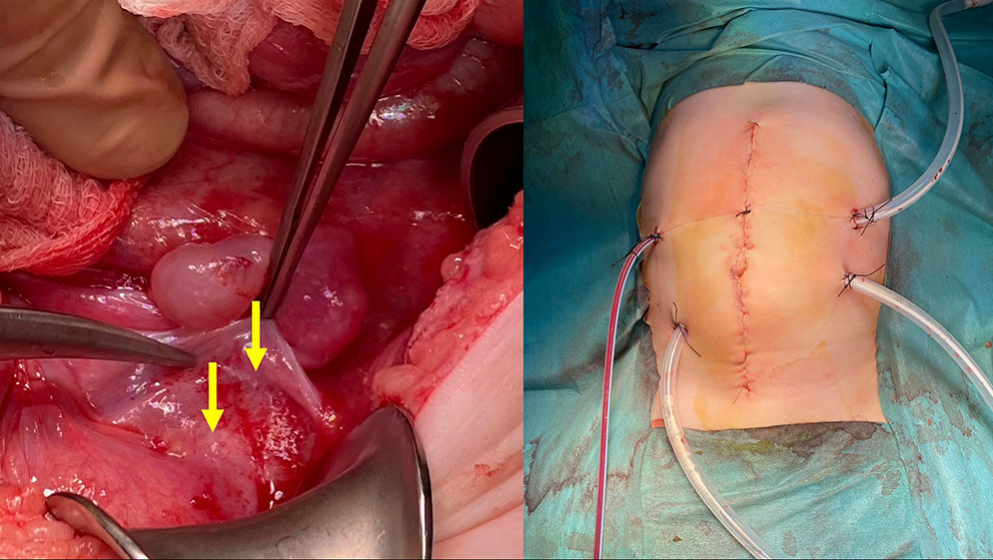
On the left—intraoperative image: pelvic peritoneum with tumor implants (yellow arrows). On the right—abdomen during HIPEC.

A 5-month-old infant presented to the emergency department due to the abdominal distention and blood in the stool. The Computed Tomography (CT) revealed a solid-cystic mass (113 × 98 × 103 mm) in the right abdominal area (Figure 3). In laboratory tests an increased C-reactive protein level was observed (40.9 mg/dL). Other parameters (blood count, asparate transaminase, alanine transaminase, alpha-fetoprotein, lactate dehydrogenase, neuron-specific enolase) were all within the age norm. Due to the unknown character of the tumor, the child underwent laparotomy elsewhere. A cecum tumor and numerous peritoneal metastasis were found intraoperatively. Primary tumor with cecum and all visible metastasis (in peritoneum and greater omentum) were removed and ileo-colonic anastomosis was performed. Postoperative course was uneventful and the child was discharged from the hospital on the day 11 after surgery without complications. Pathological examination revealed a non-RMS neoplasm—ALK1-positive Inflammatory Myofibroblastic Tumor (IMT). Two weeks after the surgery, the control MRI did not reveal any pathological lesions. Due to the presence of numerous intraperitoneal metastases during the first surgery the patient was qualified to HIPEC procedure without neoadjuvant chemotherapy. An exploratory laparotomy was performed before to proceed with HIPEC. Several peritoneal metastasis were removed from vesico-uterine pouch, pouch of Douglas and total pelvic peritonectomy was performed (Figure 4). At the end of the CRS, the CR was complete (CR0) (Figure 1). After the CRS procedure the patient underwent subsequently modified HIPEC procedure. To avoid overheating of the infant, the intraperitoneal normothermic chemoperfusion was performed in 30 min. Due to the low weight of the infant a modified dosage (9.2 mg) of intraperitoneal Doxorubicin was used. The child underwent standard postoperative chemotherapy (CWS-Guidance 2014) and received crizotinib therapy. At 12 months follow-up since treatment completion the patient remains in complete remission. To our knowledge this is the youngest patient, the only infant and the only pediatric patient with IMT who underwent the modified HIPEC procedure in the world.

## Discussion

The first pediatric reports on CRS and HIPEC was presented by Hayes-Jordan et al. in 2015 and 2018 (13, 25). According to her study CRS + HIPEC may be the most effective in children with desmoplastic small round cell tumor (DSRCT), although other histology was also admissible. According to our knowledge there are no pediatric IMT cases treated with HIPEC reported in the literature. The gold standard for IMT is surgical treatment, although chemotherapy and radiotherapy are feasible alternatives to surgery. Tao et al. (26) presented a case successfully additionally treated with non-steroid anti-inflammatory drugs (diclofenac sodium). Steroids have also been reported to be effective, especially for IMT containing IgG4SD features (27). On the other hand, the clinical trial on crizotinib administration combined with surgical treatment for ALK-positive patients resulted in complete remission in most of the cases (28). The European pediatric Soft Tissue Sarcoma Study Group (EpSSG) has recently summarized their experience in treating IMT proving its high response to chemotherapy (especially vinblastine and low-dose methotrexate) and suggested the usage of targeted inhibitors in the standard of care (29). The principal problem in the treatment of peritoneal tumors with neoadjuvant or adjuvant chemotherapy is the limited drug absorption throughout the physiological peritoneal plasma barrier (30). In such cases local application of cytotoxic drugs seems to play an important role. Intraperitoneal chemotherapy ensures a high concentration of the drug in the peritoneal cavity and reduces its systemic side effects. It should be emphasized that the macroscopic excision of all visible lesions (CRS) is crucial for the positive effect of the therapy due to the limited penetration of cytotoxic drugs into the tissues (~1 mm) (31). The time from surgery to administration of peritoneal chemotherapy is also important. HIPEC procedure in combination with CRS ensures better penetration of the drug (before the healing processes and formation of fibrin and adhesions start). Due to all of that, in the presented case CRC+HIPEC combined with postoperative chemotherapy and crizotinib seemed to be the best treatment option.

Identifying appropriate dosing regimens for the treatment of neonates and infants with cancer is a significant challenge in pediatric oncology. Most anti-cancer drugs given to children are dosed using only body surface area (BSA). However, infant's development differs significantly from older children. Thus, the cytotoxic drugs dosage should be different. In 2017 Balis et al. (32) described a modified infant chemotherapy dosing, calculated using not only BSA, but also developmental milestones. Following the above recommendations the dosage of Doxorubicin for the presented case was calculated.

Since abdominal location of IMT and its peritoneal spread is very rare and there are no more cases treated with HIPEC described in the literature no definitive conclusions can be made. Further studies involving larger patients groups are needed.

## Conclusions

CRS and HIPEC is technically possible also in infants. For its safe course patients selection and technique modification are necessary. In the world literature the best HIPEC outcome was experienced in the treatment of DSRCT, but it should be also considered in intraperitoneally disseminated IMT. A complete cytoreductive surgery preceding HIPEC directly seems to be the key factor in survival.

## Data Availability Statement

The original contributions presented in the study are included in the article/supplementary material, further inquiries can be directed to the corresponding authors.

## Ethics Statement

Written informed consent was obtained from the participant's next of kin for the publication of this case report. Written informed consent was obtained from the participant's next of kin for the publication of any potentially identifiable images or data included in this article.

## Author Contributions

HG, MM, TJ, KP-W, WB, KS, WG, EI-S, and PC: substantial contributions to the conception or design of the work, the acquisition, analysis, or interpretation of data for the work, provide approval for publication of the content, and agree to be accountable for all aspects of the work in ensuring that questions related to the accuracy or integrity of any part of the work are appropriately investigated and resolved. HG, TJ, and PC: drafting the work or revising it critically for important intellectual content. All authors contributed to the article and approved the submitted version.

## Conflict of Interest

The authors declare that the research was conducted in the absence of any commercial or financial relationships that could be construed as a potential conflict of interest.

## Publisher's Note

All claims expressed in this article are solely those of the authors and do not necessarily represent those of their affiliated organizations, or those of the publisher, the editors and the reviewers. Any product that may be evaluated in this article, or claim that may be made by its manufacturer, is not guaranteed or endorsed by the publisher.
